# Steric and Conformational Effects in Molecular Junctions

**DOI:** 10.1002/asia.202401831

**Published:** 2025-02-27

**Authors:** Yuya Tanaka

**Affiliations:** ^1^ Laboratory for Chemistry and Life Science Institute of Integrated Research Institute of Science Tokyo 4259 Nagatsuta, Midori-ku Yokohama 226-8501 Japan; ^2^ School of Materials and Chemical Technology Department of Chemical Science and Engineering Institute of Science Tokyo 4259 Nagatsuta, Midori-ku Yokohama 226-8501 Japan

**Keywords:** Break junction, Conformation, Molecular junction, Mechanoresistivity, Steric effects

## Abstract

Molecular junctions, which comprise molecules bridging two electrodes, have emerged as valuable tools for investigating the conducting properties of molecules. Although electronic effects have been extensively investigated in molecular junctions, steric and conformational effects remain underexplored. Substituents, such as long alkyl chains, are frequently introduced into molecular backbones to facilitate synthesis or improve solubility. However, their effects on conductance and junction structure are frequently overlooked. Most junction studies have focused on rigid rod molecules; however, the conductance of flexible molecules can be modulated through conformational changes. The active harnessing of this structural flexibility enables effective conductance control. This review article discusses steric and conformational effects in molecular junctions, in terms of substituent effects, conformational dynamics, and mechanical manipulation, to elucidate their roles in molecular conductance.

## Graphical abstract

This review article discusses steric and conformational effects in molecular junctions, focusing on substituent effects, conformational flexibility, and mechanical manipulation, to understand the mechanism of the impact of these factors on molecular conductance.

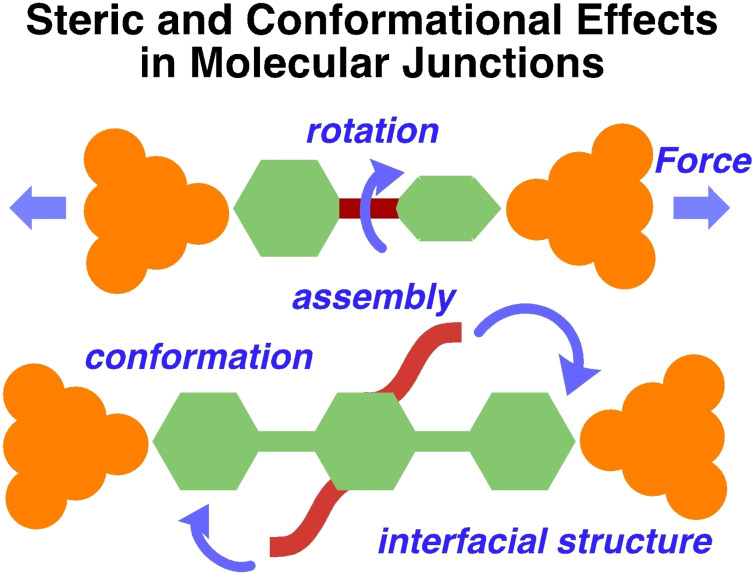

## Introduction

Single‐molecule electronics have recently attracted considerable attention because of the rapid development of nanoscience and nanotechnology.[^1^, ^2^, ^3^, ^4^] Dissimilar to rigid inorganic devices, molecular devices are unique owing to the inherent flexibility of molecules. Consequently, the steric and conformational effects of molecular junctions are some of the most important factors in molecular electronics. However, they have received considerably less research attention than electronic effects.[^5^, ^6^, ^7^] Single‐molecule conductance measurements of molecular junctions, i. e., metal‐molecule‐metal structures, have been predominantly conducted using the break junction (BJ) technique. In this approach, the electrode gap is mechanically controlled, enabling the repeated formation and rupture of molecular junctions to cycles during measurements. Two major BJ techniques are widely employed: the scanning tunneling microscope BJ (STM‐BJ) and mechanically controlled BJ (MCBJ). A detailed description of their setup has been reported.[^8^, ^9^, ^10^] During these measurements, molecular and interfacial structures fluctuate, providing rich stereochemical information. However, the data are typically accumulated and subjected to statistical analysis, and individual molecular dynamics observed in single experiments are frequently overlooked. Furthermore, studies on molecular junctions frequently emphasize the structure–conductance relationship to investigate fundamental and functional properties. This focus favors the use of “rigid rod” molecular frameworks to minimize undesired conformational effects, which can complicate conductance interpretations. In certain reports on molecular junctions, substituents embedded in molecular structures are omitted from figures in the main text. This is probably because of the assumption that they are solely introduced to fulfill synthesis requirements and improve solubility and do not significantly influence junction properties. Similarly, such substituents are frequently neglected in theoretical calculations, mainly to save calculation cost, further reinforcing this bias.

**Figure 1:**
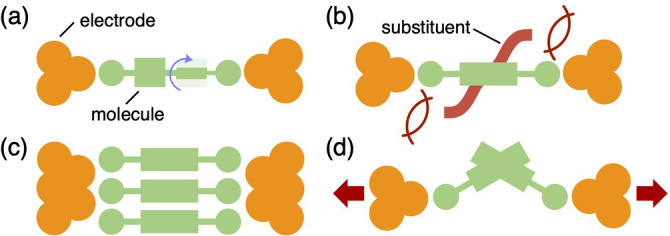
Schematic representation of steric and conformation effects in molecular junctions. (a) Conformational changes. (b) Steric effects on interfacial structures. (c) Steric effects on molecular assemblies. (d) Mechanical manipulations.

This short review article highlights several examples that challenge the aforementioned conventional approaches (Figure 1). Specifically, this paper discusses (1) how conformational changes in molecules affect conductance, (2) how substituents affect molecule–electrode interfaces, (3) molecular assemblies, and (4) how the mechanical manipulation of nanogaps enables the control of molecular junction features. Through these examples, the profound impact of subtle steric and conformation effects on molecular junction properties is illustrated.

## Conformation of π‐Conjugated Frameworks

**Figure 2:**
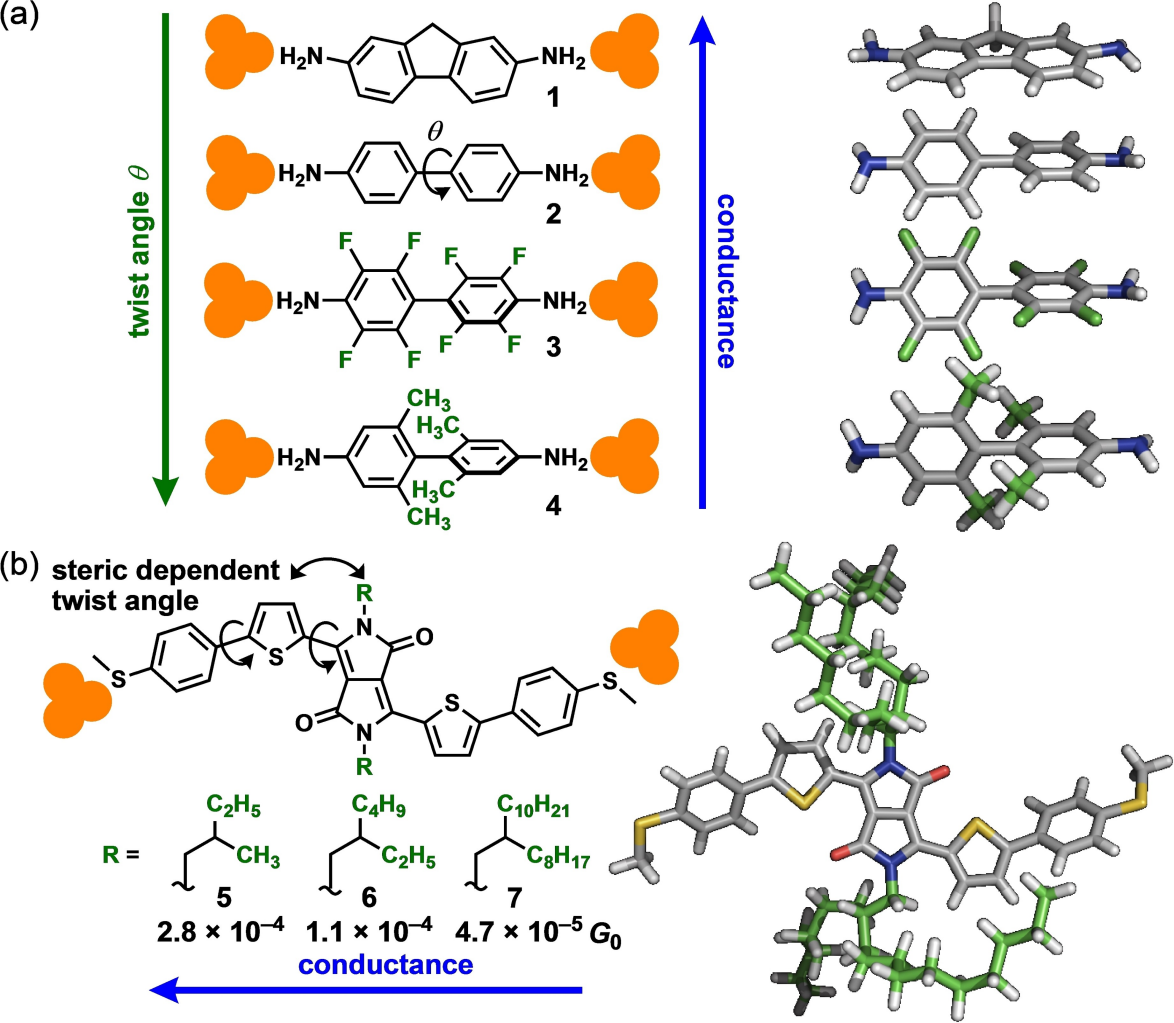
(a) Angle‐dependent conductance of biaryl compounds, including the molecular structures and optimized structures of **1**–**4**. (b) Side‐chain length‐dependent conductance in diketopyrrolopyrrole junctions, showing molecular structures of **5**–**7** and the optimized structure of **5**.

Biaryl structures are prevalent in π‐conjugated molecules, and the dihedral angles between both aromatic rings significantly influence the physicochemical properties of biaryl compounds. An early example, demonstrated by Venkataraman et al., highlights the importance of conformations using simple biaryl compounds.^[11]^ They investigated biaryl compounds **1**–**4**, with varying twisted angles controlled by connecting two rings or introducing substituents, in an STM‐BJ study (Figure 2a). The conductance of such compounds varied dramatically, spanning a couple of orders of magnitude (10^−5^–10^−3^ *G*_0_, where *G*_0_ is the quantum conductance of 77.5 μS), depending on the substituents incorporated into the backbone. The planar methylene‐linked biaryl **1** exhibited the highest conductance, whereas the fully twisted *ortho*‐methylated biaryl **4** exhibited the lowest conductance. Although the rotation rate of the biaryl compounds was sufficiently rapid relative to the measurement timescale, the dihedral angles calculated for each structure explain the observed trend in carrier transport efficiencies.

Molecule conformation can be effectively controlled by remote substituents. Zhang et al. conducted an STM‐BJ study on diketopyrrolopyrrole junctions with various long, branched alkyl side chains (**5**–**7**, Figure 2b).^[12]^ Despite having the same π‐conjugated backbone, the conductance was dependent on the side chains. The diketopyrrolopyrrole junction **5** exhibited approximately threefold and sixfold the conductances of **6** and **7**, which have longer alkyl side chains. Density functional theory (DFT) calculations revealed that as the side‐chain extended, it was hard for the π‐conjugated backbones to maintain planarity because of interactions between the side chains and backbone.

**Figure 3:**
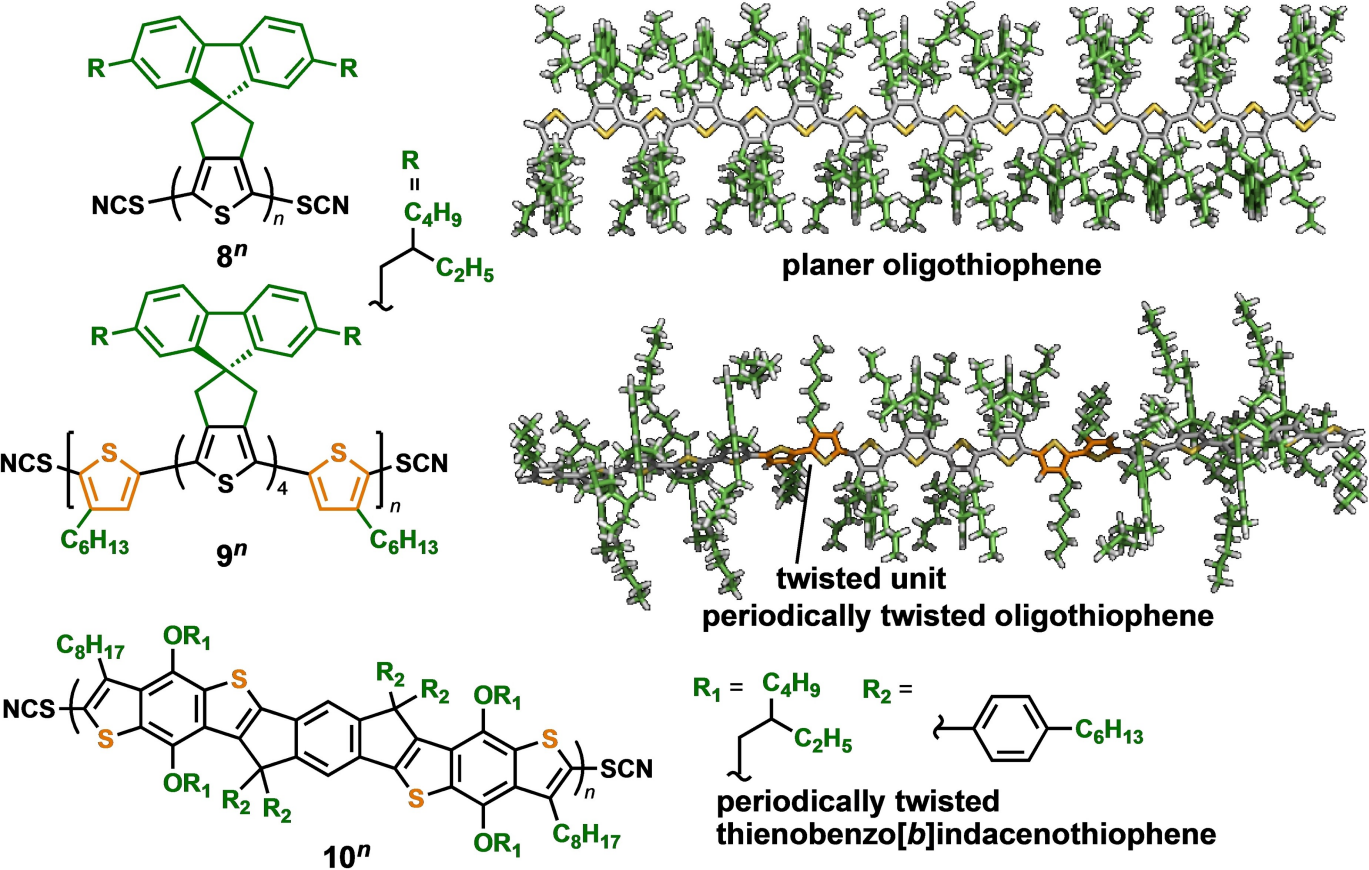
Oligothiophenes **8** ^***n***^ and **9** ^***n***^ functionalized with bulky cyclopentene units and their model structures, and fused thienobenzo[*b*]indacenothiophene oligomers **10** ^***n***^.

To develop molecular devices, it is crucial to control the conductance mechanism. At short distances, charge carriers travel through the junction *via* a coherent tunneling mechanism, whereas an incoherent hopping process dominates at long distances. However, the precise control of the crossover point, the transition from tunneling to hopping, remains unclear. Ie et al. demonstrated the control of the crossover point in oligothiophene oligomers through the steric effect.^[13]^ They designed cyclopentene‐annelated oligothiophenes, **8** ^***n***^ (*n*=2–24), with orthogonally fused bis(2‐ethylhexyl)fluorene groups (Figure 3). This unique sterically bulky functionalization rendered the thiophene oligomers planar. A molecular‐length‐dependent STM‐BJ study of **8** ^***n***^ revealed a distinct conductance decay that transitioned at repeating units 8–12, indicating that the crossover point was between them. This point was considerably shorter than that in related oligothiophene derivatives with disordered structures. The shortened crossover point was attributed to the small highest occupied molecular orbital (MO)–lowest unoccupied MO gap and reduced reorganization energy resulting from the planar structure. Furthermore, the combination of fluorene‐functionalized thiophene oligomers with twisted bithiophene units led to the design of a periodically twisted oligothiophene, **9** ^***n***^ (*n*=1–5).^[14]^ The planar hexathiophene unit is sufficiently large to stabilize radical cationic species, which are transiently generated through a hopping mechanism. In a temperature‐dependent STM‐BJ study, the thiophene 12‐mers **9^2^** exhibited a typical Arrhenius behavior with an activation energy of 0.15 eV, comparable to that of **8^12^** without twisted units. Molecular‐length dependence studies revealed that the twisted **9** ^***n***^ exhibited higher hopping conductance than the fully planar **8** ^***n***^ oligomers, owing to their regularly segmented π‐conjugation. Recently, the same group developed π‐conjugated oligomers **10** ^***n***^, featuring planar thienobenzo[*b*]indacenothiophene frameworks.^[15]^ Incorporation of alkyl groups at the periphery of the fused thiophene rings induced a periodically twisted structure, similar to **9** ^***n***^. STM‐BJ measurements of **10** ^***n***^ revealed the highest conductance among **8** ^***n***^–**10** ^***n***^, attributed to its fully planar fused framework, which minimize reorganization energy.

## Molecule‐Electrode Interfacial Structures

**Figure 4:**
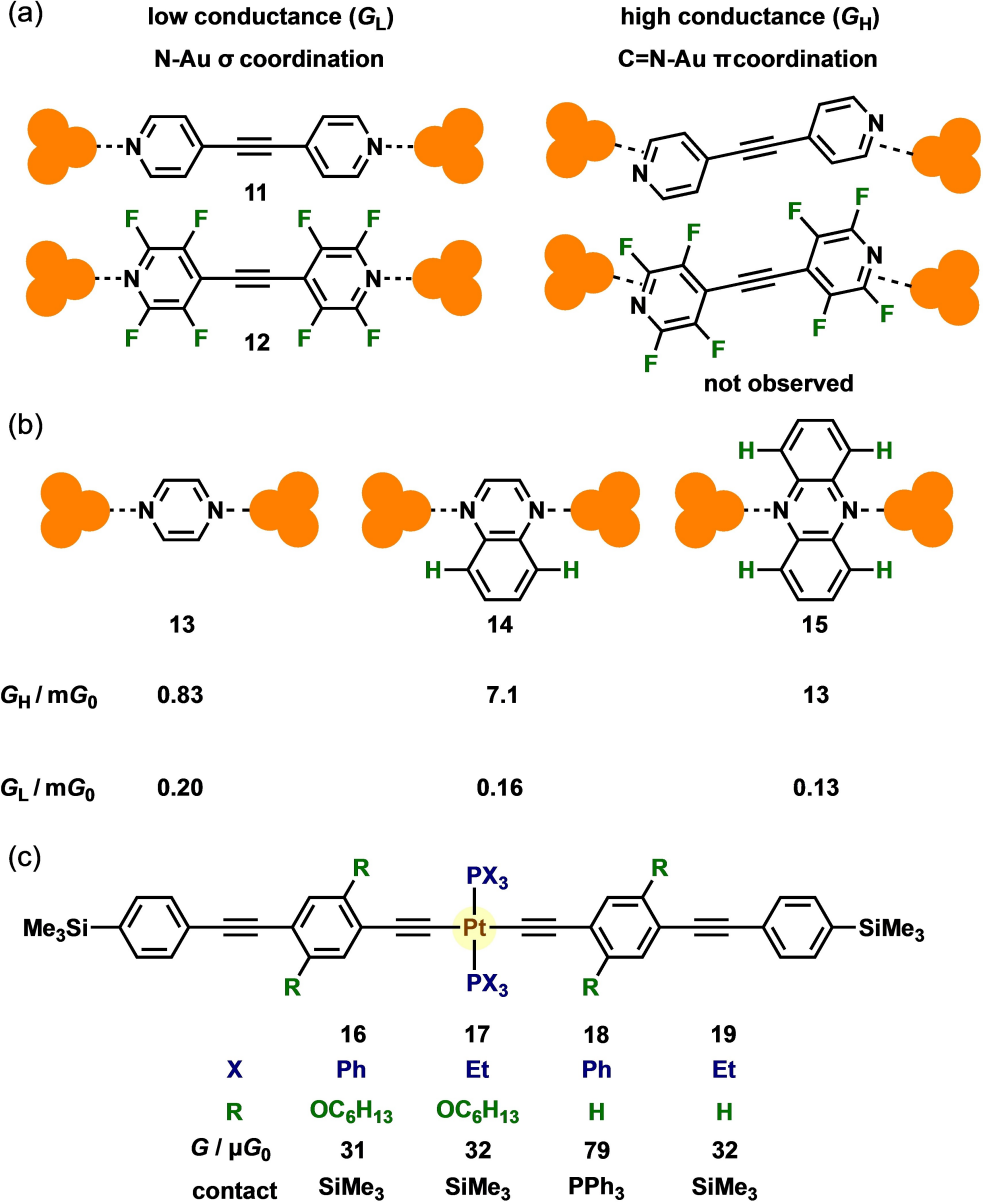
(a) Molecular junctions **11** and **12** and their two distinct binding models. (b) Molecular junctions of **13**–**15** and their conductance. (c) Molecular structures of **16**–**19**.

Structures of molecule‐electrode interfaces play critical roles in the conductance of molecular junctions. Molecular junctions frequently exhibit multiple conductances derived from different binding modes. A typical example is the well‐known thiol anchor groups featuring on‐top, bridge, and hollow structures, where the bond numbers between the thiol and electrodes are single, double, and triple, respectively.^[16]^ Although it is rather difficult to control the binding modes, the steric hindrance in molecular backbones can affect molecule–electrode interfacial structures. Smirnov et al. demonstrated an impact on simple substituents toward molecule–electrode contact. Bispyridine **11** exhibited two sets of molecular conductances, which are typically observed for bispyridine molecules (Figure 4a).^[17]^ The two sets of conductances observed for bispyridine compounds are proposed to originate from different binding models at the interface, as demonstrated in an MCBJ Raman study.^[18]^ The low conductance was derived from an N−Au σ‐type contact, while the high conductance adopts a π‐coordination of C=N to Au electrodes. However, the fluorine‐substituted derivative **12** only exhibited single, low conductance. Fluorine substituents on the alpha position of pyridine selectively hinder highly conducting binding models most probably because of steric hindrance and/or electronic perturbation.

Fujii et al. investigated the conductance behavior of pyrazine **13** and its π‐expanded derivatives, quinoxaline **14** and phenazine **15** (Figure 4b).^[19]^ These molecules exhibited two distinct conductance states, attributed to different binding models, similar to those observed in bispyridine molecular junctions. With an expansion in the π‐conjugation, the high conductance (*G*_H_) increased, whereas the low conductance (*G*_L_) decreased. To determine the origin of this trend, *I*−*V* measurements were performed to extract two parameters: *ϵ* (the energy difference between the Fermi level and frontier MOs) and *Γ* (the electronic coupling between the metal electrodes and a single molecule). In the high‐conductance state, *ϵ* decreased and *Γ* increased with an expansion in the π‐conjugated system. Conversely, in the low‐conductance state, *ϵ* and *Γ* decreased with an increase in the π‐conjugation size. For the larger compounds **14** and **15**, hydrogen atoms near the nitrogen atoms hinder metal–molecule interactions at the junctions, leading to reduced *Γ*. This steric effect was more pronounced in the low‐conductance σ‐type binding mode than in the π‐type binding mode.

Low et al. reported the steric‐dependent junction formation of platinum acetylide complexes bearing two phosphine and diethynylbenzene‐based ligands with/without long alkoxy groups (R, Figure 4c).^[20]^ An STM‐BJ study of Pt acetylide complexes **16** and **17**, which bear alkoxy groups, revealed similar conductances of approximately 3×10^−5^ *G*_0_, regardless of the phosphine ligands (X=Ph or Et). Two‐dimensional (2D) histograms showed stretch lengths corresponding to the molecular length, indicating terminal contacts at trimethylsilyl (TMS) groups. When the long alkoxy groups were replaced with hydrogen atoms, complex **18** exhibited a relatively high conductance (X=Ph, ~8×10^−5^ *G*_0_), dissimilar to that of **19** (X=Et) which was similar to that of **16**. In the 2D histogram for **18**, a shorter stretch length was observed, suggesting that junction formation occurred at the PPh_3_ groups instead of the TMS groups. These results show that long alkoxy groups effectively suppress undesired junction formation at PPh_3_ groups through steric hindrance.

**Figure 5:**
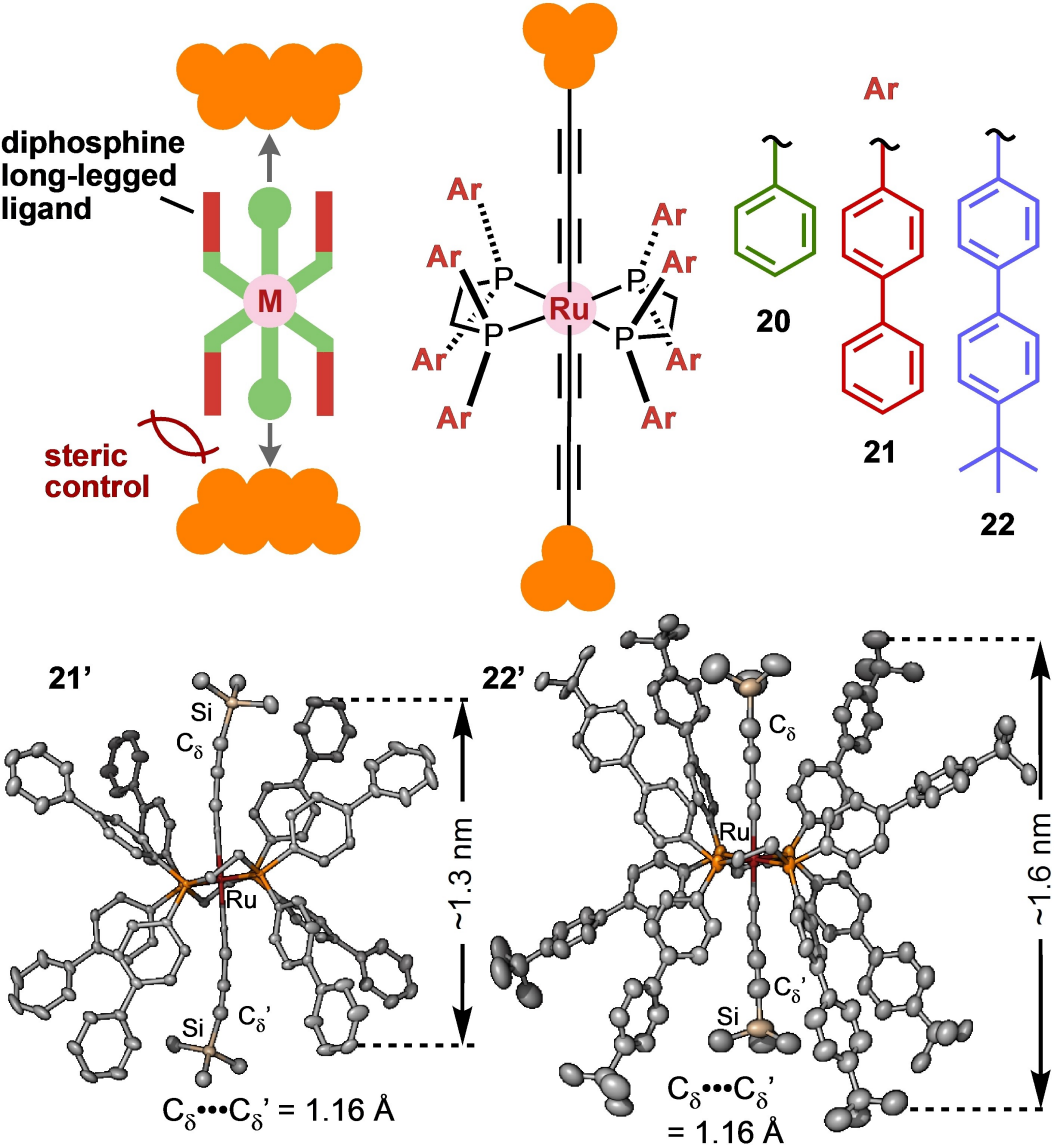
Molecular design and structures of **20**–**22** featuring long‐legged phosphine ligands, with the solid‐state structures of precursors **21′** and **22′**.

By actively incorporating the steric effect, the binding models of molecular junctions can be controlled. Tanaka et al. attempted to develop ligand‐controlled interfacial structures of molecular junctions using ruthenium acetylide complexes.^[21]^ Acetylide anchor groups adopt three binding models (on‐top, bridge, and hollow).^[22]^ The team designed ruthenium complexes with long, bulky substituents on phosphine ligands, such as biphenyl **21** and *tert*‐butylbiphenyl groups **22**. The X‐ray crystallography of their precursors **21′** and **22′** revealed that the long‐legged ligands were sufficiently extended to perturb surface structures during molecular junction formation (Figure 5). A surface‐enhanced Raman scattering study of self‐assembled monolayers on Au substrates revealed well‐defined, sharp signals for complexes **21** and **22** compared with those of **20**, which featured less bulky phenyl rings.^[23]^ DFT analysis indicates that the on‐top binding mode was selectively adopted by the bulky **22** because of steric repulsion between the electrodes and ligands. STM‐BJ experiments further demonstrated the effects of the bulky ligands. The full width at half maximum obtained from 1D histograms for **21** and **22** were 1.6‐ and 3.6‐fold narrower, respectively, than that of **20**, whereas their conductance was virtually identical. This suggests that the bulky ligands restricted the binding angles of the molecular junctions and selectively promoted the on‐top binding mode during junction formation.

## Molecular Assemblies

**Figure 6:**
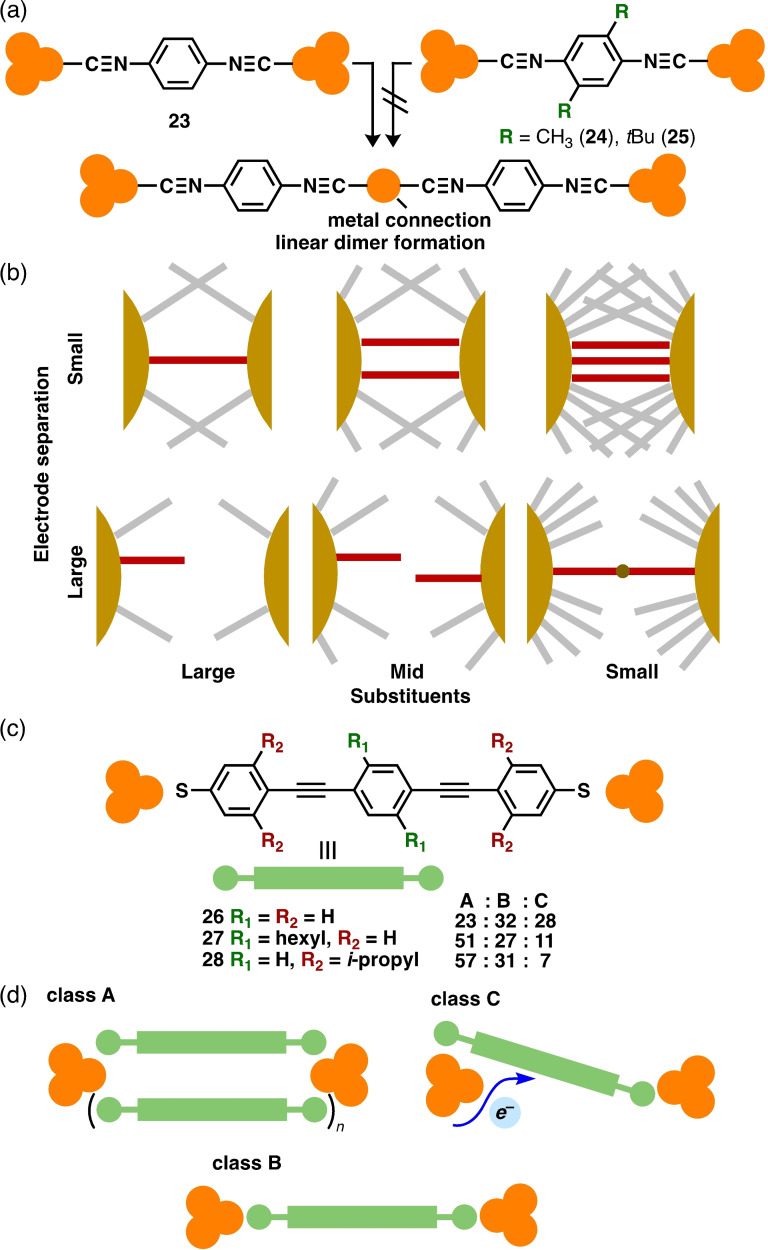
(a) Molecular junctions of **23**–**25** and the formation of metal‐connected dimers. (b) Substituent‐ and electrode‐separation‐dependent junction formation. (c) Molecular junctions **26**–**28** and their class ratios. (d) Schematic representation of possible structures of classes A–C.

BJ studies provide valuable insights into molecular assemblies, such as multiple parallel junctions or oligomerization. Although the formation of molecular assemblies is frequently controlled by concentration and solvents, substituents offer a method of regulation. Calame et al. investigated steric effects on the in situ formation of 1D coordination polymers using the MCBJ technique (Figure 6a).^[24]^ When 1,4‐diisocyanobenzene **23** was investigated in mixed tetrahydrofuran/mesitylene solutions at a concentration of 100 mM, two distinct conductance features were observed (8×10^−3^ and 2×10^−4^ *G*_0_ at lengths of 6.4 and 9.0 Å, respectively). In comparison with related studies, the authors attributed the higher conductance to multiple parallel molecular junctions and the lower conductance to linear dimer formation with a single Au atom connection. The incorporation of methyl substituents into a phenylene ring (**24**) eliminated the low‐conductance signal, indicating the suppression of linear dimer formation. The introduction of bulky *tert*‐butyl substituents into **25** resulted in a single conductance peak at 5×10^−4^ *G*_0_ with a short length. This was attributed to single‐molecule junctions, as the formation of multiple parallel junctions was prevented by the bulky substituents. This phenomenon could be explained by a reduction in the local density in the junctions due to the sterically demanding substituents (Figure 6b). When molecules are densely packed between electrodes, the probability of forming parallel junctions and linear dimers after reacting with Au electrodes increases. This behavior parallels observations made under concentrated/diluted conditions. Thus, simple alkyl substituents can effectively control molecular assemblies in junctions.

Similar substituents but different observations were reported by Mayor et al., who investigated the steric effects of phenylene ethynylene molecules **26**–**28** (Figure 6c).^[25]^ Non‐substituted **26**, a well‐investigated system in BJ studies; **27**, which bears additional hexyl groups on the central phenylene rings; and **28**, which bears isopropyl groups on the terminal phenylene rings, were subjected to an MCBJ study in a 5 μM CH_2_Cl_2_ solution. A relatively broad conductance ranging from 10^−3^ to 10^−6^ *G*_0_ was observed. Using a reference‐free unsupervised clustering algorithm, the data were classified into three major groups (A, B, and C) based on conductance, in the order of A>B>C. The compounds in classes B and C exhibited similar features, whereas in class A, conductance decay was observed prior to the rupture of the junctions. According to the existing literature, classes A and B were designated as multiple‐ and single‐molecule junctions, respectively (Figure 6d). Class C was assumed to be derived from the junction where an electron is injected from phenylene or acetylene linkers. The contribution of class A increased by incorporating alkyl groups (relative ratio (%): **26**, 23; **27**, 51; **28**, 57). Furthermore, the conductance of class A followed the order of **28** (4.3×10^−4^ *G*_0_)>**27** (3.0×10^−4^ *G*_0_)>**26** (2.6×10^−4^ *G*_0_). This suggested that the number of bridging molecules was dependent on the alkyl substituents. The enhanced assembly by alkyl groups was likely due to weak electrostatic interactions (London dispersion forces) and van der Waals interactions. This study provides a unique example of how alkyl substituents function as molecular “glue” to promote assembly.

## Mechanoresistivity

**Figure 7:**
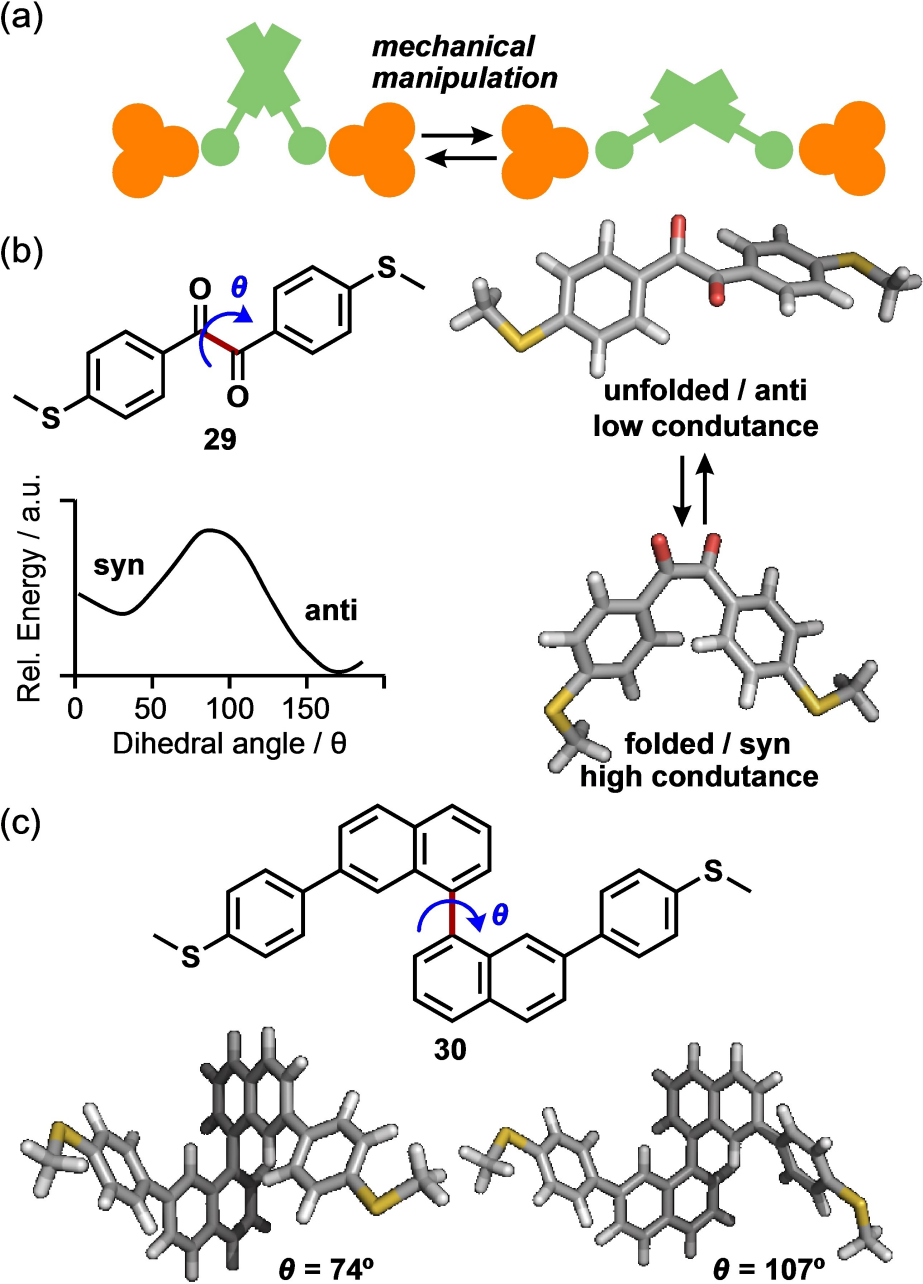
(a) Mechanical manipulation of a molecular junction. (b) Anti and syn conformations of **29**. (c) Molecular structure of **30** and its optimized structures with different dihedral angles (*θ*).

The mechanical manipulation of nanogaps enables the dynamic switching of the conductance without the chemical modification of molecular backbones (Figure 7a). This mechanoresistivity (change in conductance through mechanical manipulations) has recently attracted significant attention.^[26]^ As mentioned above, most molecules investigated in BJ experiments utilize rigid, rod‐like π‐conjugated frameworks to achieve high and well‐defined conductance. Contrarily, diketone **29** adopts folded (syn) and unfolded conformations (anti) because of its flexible carbon–carbon single bond (Figure 7b). These conformations can easily interconvert during BJ measurements. Vezzoli et al. performed STM‐BJ experiments on diketone **29**, which exhibits two distinct conductance peaks corresponding to its folded and unfolded conformations.^[27]^ Mechanical manipulation, achieved by modulating the molecular junction distance (0.4 nm), led to an increase in the conductance by a factor of as high as 21 upon compression. In comparison, a stilbene derivative and an asymmetrical molecule with a single aryl carbonyl group revealed only twofold and threefold increments under the same conditions. Power spectral density analysis revealed that the high conductance attributed to the folded conformation originated from a through‐space transport mechanism between two aryl rings.

The same group further investigated the mechanoresistivity of binaphthyl compound **30** (Figure 7c)^[28]^ in an STM‐BJ study. Two prominent conductance peaks observed at 10^−3^ and 10^−5^ *G*_0_ were ascribed to the cisoid and transoid conformations, respectively. Piezo‐modulation experiments confirmed the mechanoresistivity of **30**, showing alternating high and low‐conductance cycles as the tip distance in the junction was modulated. Smooth and continuous conductance changes were observed when the modulation amplitude was sufficiently large. DFT‐based transport calculations revealed that the drastic change in conductance in the cisoid configuration originated from the formation of a conductance pathway through the π‐orbital of two naphthyl groups.

**Figure 8:**
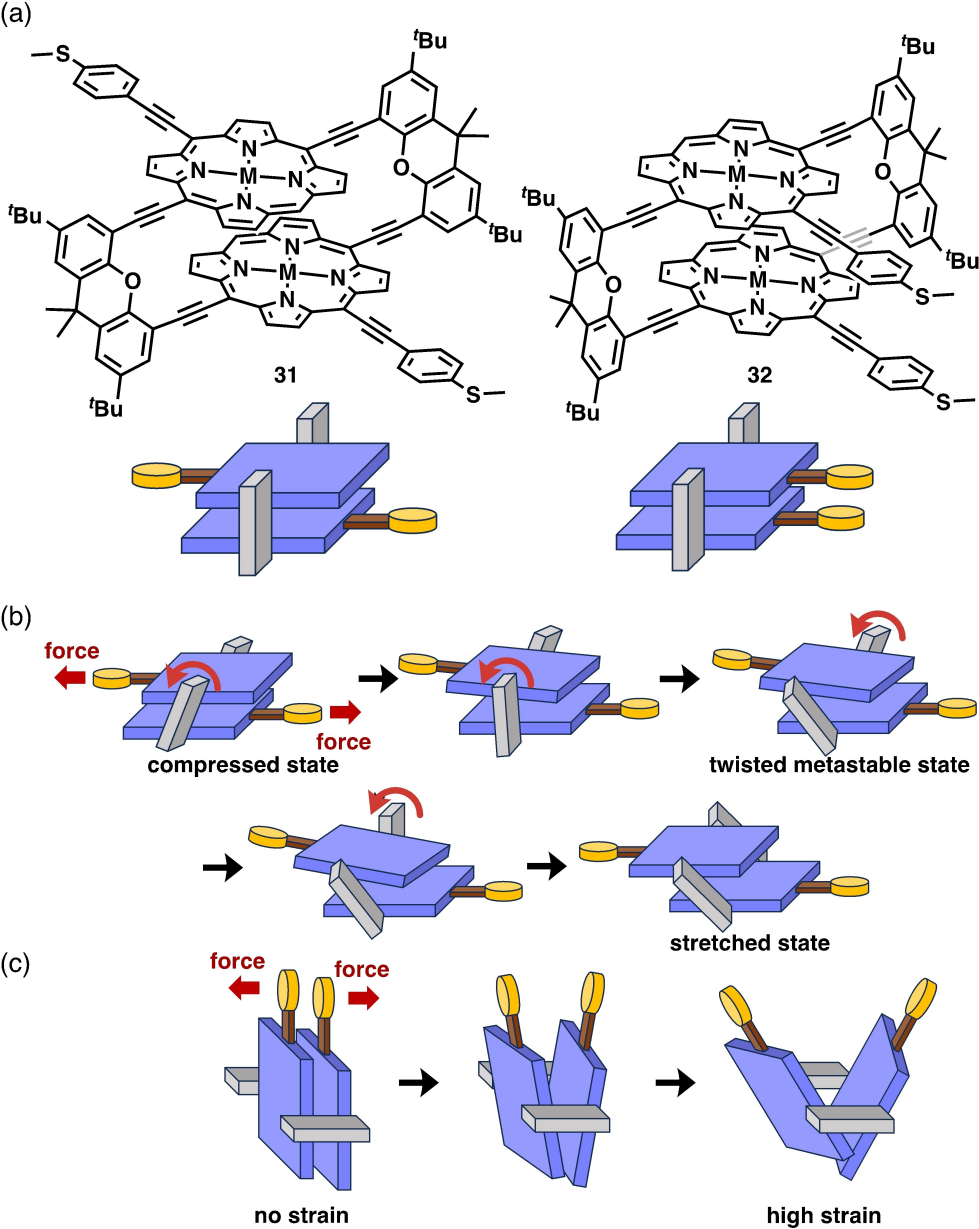
(a) Molecular structures of **31** and **32**. (b) Schematic representation of the stepwise transition of **31** from the compressed state to the stretched state. (c) Force‐induced strain of **32** during BJ experiments.

Mayor et al. designed unique porphyrin‐based cyclophanes **31** and **32** as new mechanoresistive molecular systems, which exhibited unique conductance behaviors during MCBJ measurements (Figure 8a).[^29^, ^30^] Cyclophane **31**, featuring two porphyrin faces in a *trans* conformation connected by two diethynyl xanthene linkers, adopts two conformers (compressed and stretched states) dependent on the angles of the xanthene linkers. BJ experiments revealed a conductance of approximately 2×10^−6^ *G*_0_, with a breaking distance of 2.3 nm, falling between the S−S distances of the compressed (2.1 nm) and stretched (2.8 nm) states. Multiple oscillations in the conductance, with changes of up to 1.5 orders of magnitude observed in individual traces, demonstrated the significant mechanoresistivity of **31**. The atypical oscillation behavior was supported by DFT simulations, which revealed that the xanthene linker flipped stepwise from the compressed state to the stretched state (Figure 8b). During stretching, multiple drops in the conductance were simulated, caused by destructive quantum interference through a π‐stacking porphyrin pair.

The *cis* isomer **32** also exhibited mechanoresistivity. In the BJ study, after an initial decrease in conductance with an increase in the nanogap distance, a sudden increase in conductance was observed. This phenomenon can be explained as follows: the initial high conductance originated from through‐space charge transport (Figure 8c). With an extension in the nanogap, the conductance decreased because of the large gap. However, the stacking distance between the two porphyrin units gradually decreased at the edge, enhancing the π‐stacking interactions and increasing the conductance. Further strain hindered π‐stacking interactions between the porphyrins, leading to a final decrease in conductance.

## Summary and Outlook

This review article highlights the underestimated steric and conformational effects in molecular junctions. These effects cause unique conductance behaviors during BJ measurements by alternating MO energy level *ϵ* and/or electronic coupling *Γ*. Even simple alkyl groups, which are frequently incorporated to improve solubility during processing, can significantly influence molecular conformation (change in *ϵ*), molecule–electrode interfacial structures (change in *Γ*), and the assembly or disassembly of molecules in junctions (change in either *ϵ* or *Γ*). Furthermore, the manipulation of nanogap distances can induce changes in molecular conformation in junctions. These examples clearly demonstrated that detailed molecular design with steric and conformational control is essential to achieve the desired functions in molecular electronics. An exploration of these effects can pave the way for the design of more efficient and tunable molecular devices, enabling advancements in nanoscale electronic applications.

## Conflict of Interests

The authors declare no conflict of interest.

1

## Biographical Information

*Yuya Tanaka received his PhD degree from Tokyo Institute of Technology in 2010 under the supervision of Professor Munetaka Akita. He also spent half a year working with Professor Claude Lapinte at Université de Rennes1 while pursuing his PhD. Thereafter, he moved to the University of Hong Kong for postdoctoral research with Professor Vivian W. W. Yam. In 2012, he returned to Tokyo Tech to work with Professor Takafumi Ueno as a JSPS postdoctoral research fellow. In 2013, he accepted his current position as an assistant professor at the same institute. Tokyo Tech and Tokyo Medical and Dental University have merged on October 1, 2024, to form Institute of Science Tokyo*.

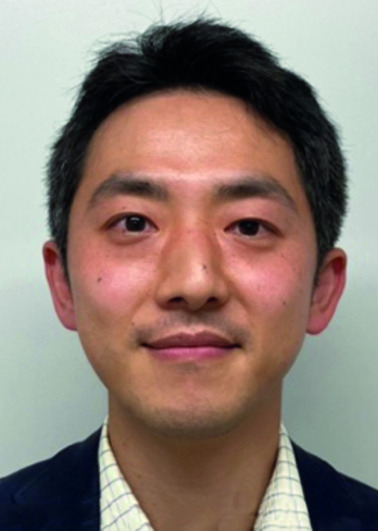

## Data Availability

Data sharing is not applicable to this article as no new data were created or analyzed in this study.
